# Comparison of the glidescope, CMAC, storz DCI with the Macintosh laryngoscope during simulated difficult laryngoscopy: a manikin study

**DOI:** 10.1186/1471-2253-12-11

**Published:** 2012-06-21

**Authors:** David W Healy, Paul Picton, Michelle Morris, Christopher Turner

**Affiliations:** 1 Department of Anesthesiology, The University of Michigan, 1500 E. Medical Center Dr., 1H247, SPC 5048, Ann Arbor, MI, 48109-5048, USA

**Keywords:** Videolaryngoscopy, Manikin, Difficult laryngoscopy, Devices

## Abstract

**Background:**

Videolaryngoscopy presents a new approach for the management of the difficult and rescue airway. There is little available evidence to compare the performance features of these devices in true difficult laryngoscopy.

**Methods:**

A prospective randomized crossover study was performed comparing the performance features of the Macintosh Laryngoscope, Glidescope, Storz CMAC and Storz DCI videolaryngoscope. Thirty anesthesia providers attempted intubation with each of the 4 laryngoscopes in a high fidelity difficult laryngoscopy manikin. The time to successful intubation (TTSI) was recorded for each device, along with failure rate, and the best view of the glottis obtained.

**Results:**

Use of the Glidescope, CMAC and Storz videolaryngoscopes improved the view of the glottis compared with use of the Macintosh blade (GEE, p = 0.000, p = 0.002, p = 0.000 respectively). Use of the CMAC resulted in an improved view compared with use of the Storz VL (Fishers, p = 0.05). Use of the Glidescope or Storz videolaryngoscope blade resulted in a longer TTSI compared with either the Macintosh (GLM, p = 0.000, p = 0.029 respectively) or CMAC blades (GLM, p = 0.000, p = 0.033 respectively).

**Conclusions:**

Unsurprisingly, when used in a simulated difficult laryngoscopy, all the videolaryngoscopes resulted in a better view of the glottis than the Macintosh blade. However, interestingly the CMAC was found to provide a better laryngoscopic view that the Storz DCI Videolaryngoscope. Additionally, use of either the Glidescope or Storz DCI Videolaryngoscope resulted in a prolonged time to successful intubation compared with use of the CMAC or Macintosh blade. The use of the CMAC during manikin simulated difficult laryngoscopy combined the efficacy of attainment of laryngoscopic view with the expediency of successful intubation. Use of the Macintosh blade combined expedience with success, despite a limited laryngoscopic view. The limitations of a manikin model of difficult laryngoscopy limits the conclusions for extrapolation into clinical practice.

## Background

Successful management of both the expected and unexpected difficult laryngoscopy is an essential component of safe medical care. Difficulty encountered during laryngoscopy is one aspect of overall difficulty in airway management. Unfortunately, current methods of airway assessment are poor screening tests for difficult laryngoscopy due to their generally low positive predictive value [1-4]. Therefore, anesthesia providers must develop and maintain skills to promptly and effectively manage unexpected difficulty encountered during laryngoscopy. Videolaryngoscopy presents a new approach for the management of difficult and rescue laryngoscopy and has the additional potential to enhance the education of novices [5-7]. Compared with direct laryngoscopy these systems allow glottic visualization without alignment of the laryngeal, pharyngeal and oral axes. It remains to be shown if this potential advantage over direct laryngoscopy, translates to an improvement in performance during true difficult laryngoscopy. There is little prospective evidence comparing the performance features of these devices with each other in this setting. Previous investigators have reported the use of videolaryngoscopy in patients at higher risk of difficulty, in obese subjects [8-11] or those with cervical spine limitation [12-19]. These subjects were at higher risk of difficult but their true difficulty remained unknown as direct laryngoscopy was not performed before use of the study device. The degree of difficulty should ideally be graded by an independent observer blinded to the study device during a previous direct laryngoscopic attempt [20,21].

The videolarygoscopes included in the current study were chosen based on similarities in form and function. They are all examples of rigid blade videolaryngoscopes thought to improve the view of the glottis based on their video capabilities. A high fidelity airway simulation manikin (AirSim Advance, Trucop, Belfast, UK) was modified to consistently reproduce a Cormack and Lehane III or IV view on standard direct laryngoscopy, where no portion of the vocal cords could be observed. A grade III or IV Cormack and Lehane view at direct laryngoscopy was defined as a difficult view in the American Society of Anesthesiologists Task Force guidelines on management of the difficult airway [1,2] and has been used as such in multiple studies.

The decision to use a high fidelity airway manikin in this prospective study, instead of patients with known difficult direct laryngoscopy, was made on ethical and technical reasons, and with the understanding that any findings would be less relevant to actual clinical care. We wanted to ensure a standardized, consistent, true difficult laryngoscopy for every laryngoscopic attempt to allow adequate device comparison. Additionally, we felt it unethical to allow our subjects to perform endotracheal intubation, using unproven videolaryngoscopic equipment laryngoscopic equipment in patients with known difficulty encountered during previous direct laryngoscopy.

The aim of this study was to investigate the performance features of a selection of video laryngoscopes in a difficult laryngoscopy scenario reproduced by a high fidelity airway manikin (Airsim Advance, Trucorp Ltd, Belfast, UK).

## Methods

This is a prospective randomized crossover study investigating the performance factors of three methods of videolaryngoscopy compared with the Macintosh blade. Approval by the Institutional Review Board (University of Michigan Ann Arbor) was sought but not required. Written informed consent to participate was not obtained as the measurements obtained were incidental to the provider’s familiarization with new equipment, the test subjects were all volunteers, and the intubations performed on manikins. Thirty anesthesia providers (subjects) participated from a single, large University Hospital. The subjects consisted of 10 Faculty members, 10 Anesthesiology Residents in Training and 10 Nurse Anesthetists in an attempt to reproduce the skill distribution of our academic anesthesia department. All providers had extensive experience with the Macintosh blade for laryngoscopy, limited experience with the Glidescope (<20 intubations) and no experience with the Storz videolaryngoscope and Storz CMAC. Immediately before the testing period all subjects were instructed in the use of all devices and allowed 10 minutes of familiarization time.

**Figure 1:**
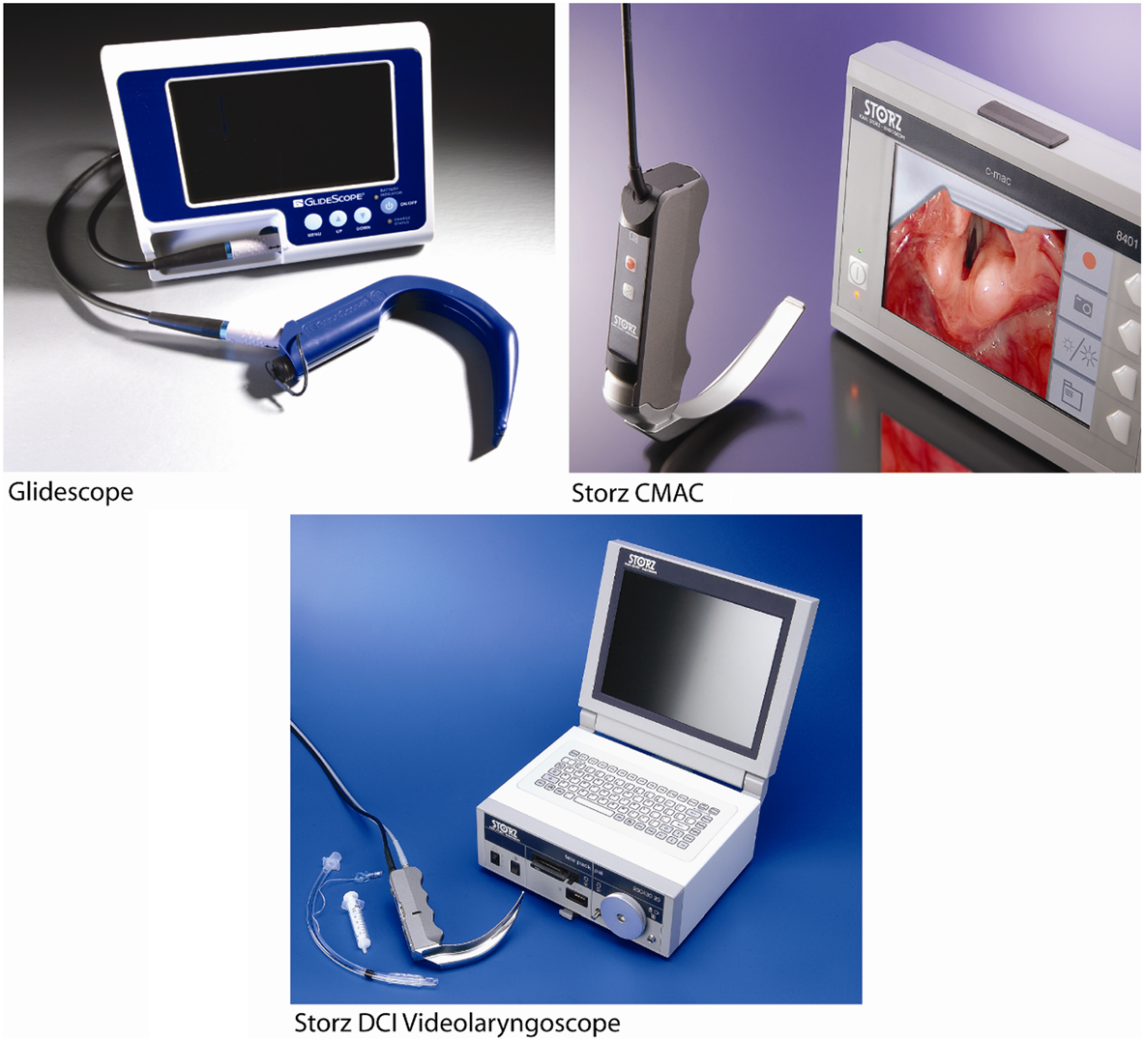
Videolaryngoscopes: Glidescope, Storz VL, and CMAC (left to right).

The following devices were compared: the Macintosh laryngoscope (Heine Optotechnik GmbH & Co. KG, Herrsching, Germany), the Glidescope Videolaryngoscope (Glidescope) (Verathon, Bothell, WA), the Storz C-MAC Videolaryngoscope (CMAC) (Karl Storz, Tuttlingen, Germany), the Storz DCI® (Direct Couple Interface) Video Intubation System (Storz VL) (Karl Storz, Tuttlingen, Germany) (Figure1).

**Figure 2:**
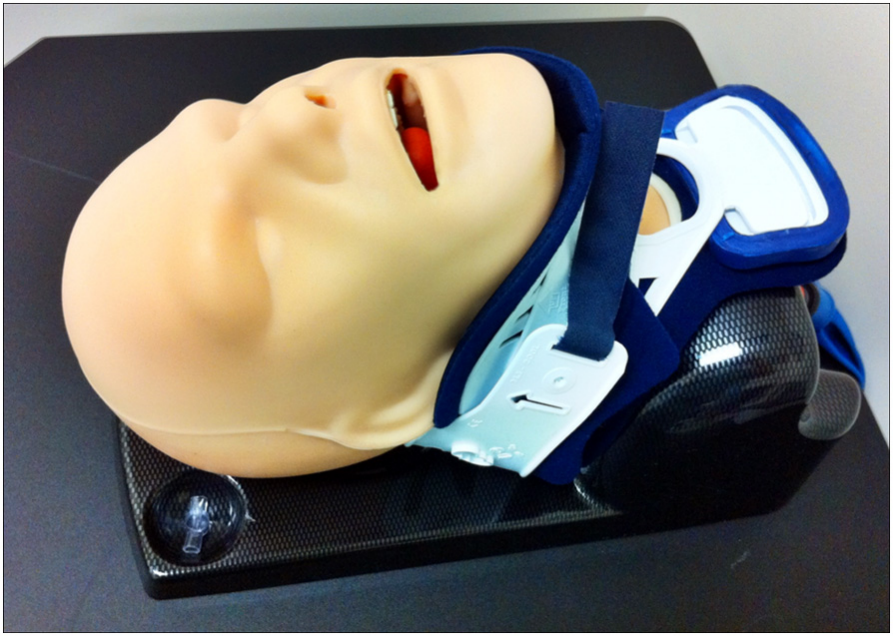
Difficult Laryngoscopy Manikin.

The subjects were randomized to attempt tracheal intubation with each of the 4 laryngoscopes. The sequence of attempts with each device and scenario was randomized with a table and random number generator. The subjects initially attempted the intubation of a normal manikin with no modifications, to familiarize the subjects with the equipment and testing protocol. They then attempted a simulated difficult laryngoscopy. The tongue was inflated with 90mls of air and a hard collar applied in the same position for each intubation attempt (Figure2). Data were collected and analyzed for the simulated difficult laryngoscopy only.

All intubations were performed with a 7 mm endotracheal tube (ETT) (Portex). A stylet was used for all intubations and preformed to a standardized curvature. Subjects were timed from the moment the laryngoscope entered the mouth until the moment the laryngoscope was removed from the mouth at the end of the intubation attempt. Following placement of the ETT subjects were asked to grade the best view of the larynx achieved, according to the Cormack and Lehane grading system. ETT position was confirmed by inflating the manikin’s lungs with air from a self-inflating bag (Mercury Medical, Clearwater, FL, USA). The primary endpoints recorded were the time taken to completion of intubation, and the occurrence of successful tracheal intubation. Failure of intubation was defined as any intubation attempt of more than 120 seconds duration or inability to successfully place the ETT into the trachea. The secondary endpoint was the best Cormack and Lehane grade view encountered during laryngoscopy.

*A priori* sample size testing was performed assuming a univariate single group repeated measures analysis of variance for the time to intubation measurements. Using an effect size of 1.4 seconds (obtained from a pilot study and literature review) and alpha level of 0.05, the sample size of 25 subjects was calculated to have a 99% power to detect the specified difference in outcome. The effect size was the variance of the estimated means between the blades, divided by a complex function of the standard deviation (estimated at 6 for each) and the correlation (weak).

The outcomes of “Time to Successful Intubation” and grade view were analyzed by statistical techniques appropriate for repeated measures; accounting for the repeated performance of tasks by the subjects potentially resulting in an improvement of performance termed the practice effect. When considering the outcome of grade view at laryngoscopy, the groups were divided in two according the view obtained: Good view (Cormack and Lehane I and II) and Poor view (Cormack and Lehane III and IV). The method of Generalized Estimating Equations (GEE) was used to test the hypothesis that there was no difference between the view obtained by the three methods of video laryngoscopy and that of the Macintosh blade. The model was performed with the Macintosh blade and then CMAC as reference blades. An estimate of within subject correlation (practice effect) was made and expressed as an r-value relating to the correlation among try (in which order a subject used a device). To identify any difference in grade view obtained between the methods of videolaryngoscopy, the data were analyzed by individual comparisons using Fishers Exact 2-sided test. To investigate the differences in Time to Successful Intubation the method of Generalized Linear Model (GLM) was used. Significance was assessed and an estimate of within subject correlation was made and expressed as an r-value. The rate of failure was analyzed by a Pearson Chi-square.

Statistical analyses were performed using SPSS 16.0, STATA was used for the GEE and GLM testing. nQuery Advisor was used for the sample size calculations. P < 0.05 was considered statistically significant.

## Results

The overall intubation metrics for each device are presented in Table 1.

**Table 1:** Intubation Metrics

	**CMAC (n = 30)**	**Storz VL (n = 30)**	**Glidescope (n = 30)**	**Macintosh (n = 30)**
Grade View (mean +/−SD)	1.4(+/−0.5)	1.6(+/−0.8)	1.7(+/−0.7)	3.0(+/−0.7)
Time to Successful Intubation (median+/−IQR)	19.2[9.3]	22.9[16.0]	36.0[24.7]	19.8[11.3]
Failures (n and %)	0(0%)	4(13%)	1(3%)	2(7%)

### Time to successful tracheal intubation (TSTI)

**Figure 3:**
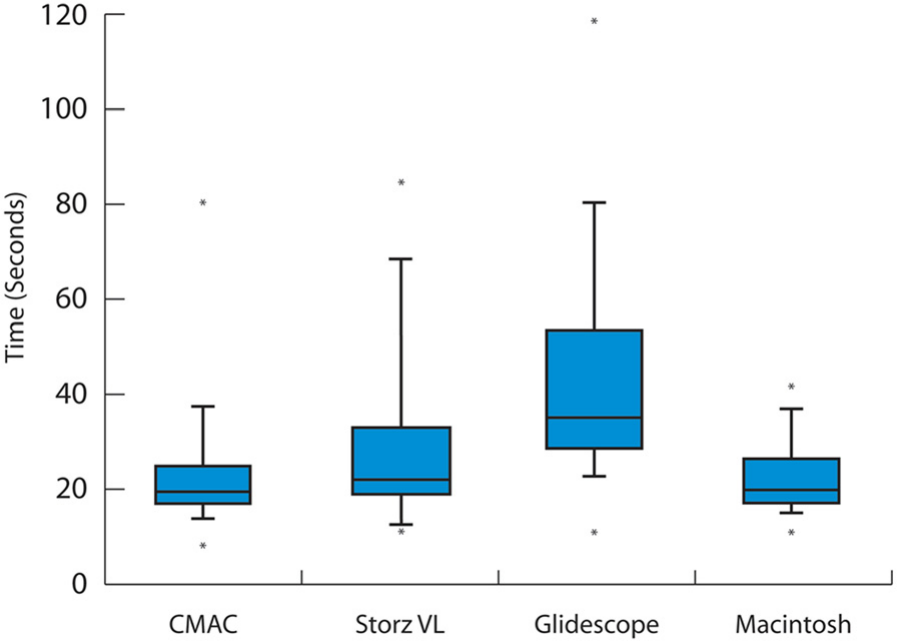
Time to Successful Intubation (Median with percentile distribution).

The times to successful intubation associated with use of the various devices are presented in Figure3. Before analysis, the failures were removed for this outcome alone. It was found that the use of the Glidescope or Storz VL resulted in a statistically significantly prolonged TTSI compared to use of the Macintosh blade (GLM, Mac reference blade, p = 0.000, p = 0.029 respectively). No statistically significant difference existed in TTSI between use of the Macintosh or CMAC blade (GLM, p 0.755). The correlation amongst try within the subjects was estimated to be weak to moderate with this comparison (r = 0.126). When the other devices were compared with the CMAC there was a significant increase in TTSI associated with use of the Glidescope or Storz VL (GLM, CMAC reference blade, p 0.001, p 0.033 respectively). The correlation amongst try within the subjects was estimated to be weak in this comparison (GLM, r = 0.049).

### Grade view at laryngoscopy

**Figure 4:**
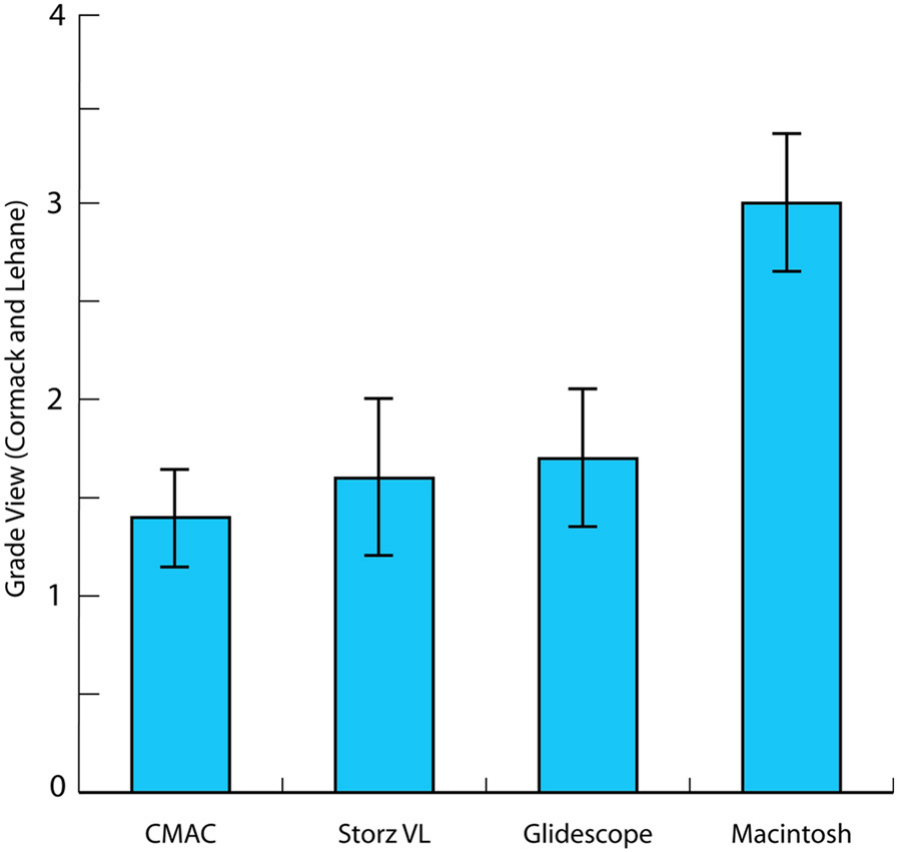
Grade view (Cormack and Lehane) Mean with error bars.

Figure4 demonstrates an inferior grade view when the Macintosh blade was used compared to all three of the methods of video laryngoscopy. It was found that subjects were much less likely to have a difficult view (C&L grades 3 and 4) with the use of the Glidescope, CMAC or Storz Videolaryngoscope compared with use of the Macintosh blade (GEE model, P = 0.000, p = 0.002, p = 0.000 respectively). There was little practice effect associated with this outcome as within subjects the correlation among try (which order the device was used) was weak (GEE, r =0.048). When the methods of videolaryngoscopy were compared with each other, the use of the CMAC resulted in a statistically significant improvement in grade view when compared with use of the Storz VL (Fishers Exact 2-sided test, p = 0.050). However, no statistically significant differences in grade view were found between the use of the other video laryngoscopes.

### Failure to intubate

**Figure 5:**
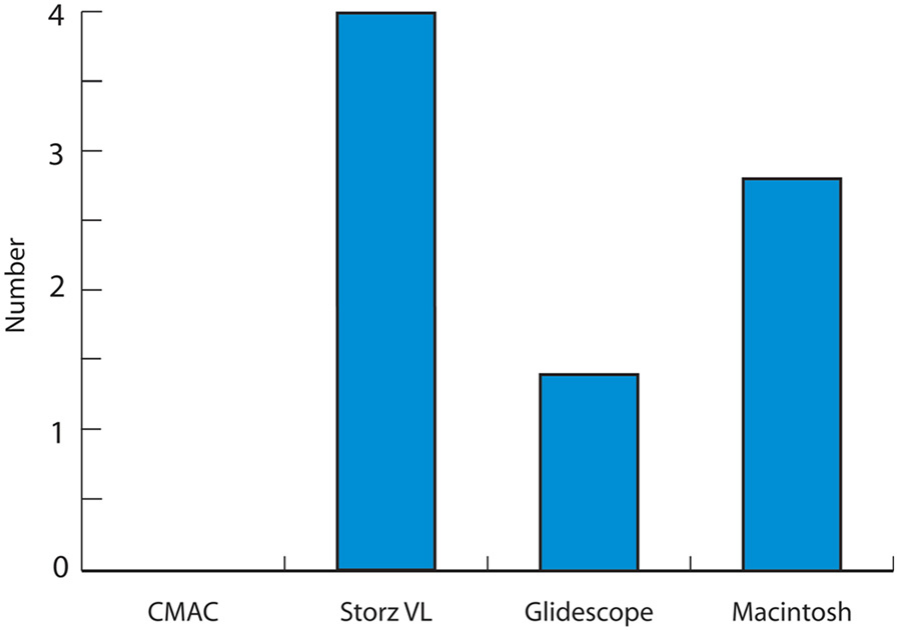
Failure to intubate (less than 120 seconds).

The numbers of intubation failures with each device is presented in Figure5. The highest number of failures occurred with use of the Storz video laryngoscope (4 failures) contrasting with no failures in CMAC use. However, no significant difference was found on statistical testing (Pearson Chi-square, p = 0.32).

## Discussion

The results of this study demonstrate that use all the video laryngoscopes improved the grade view at laryngoscopy in a simulation of difficult laryngoscopy compared with the Macintosh blade. There was a significant reduction in time to intubation of the difficult laryngoscopy manikin when the CMAC or Macintosh blade was used for intubation compared with the Glidescope or Storz Video laryngoscope.

An improvement of glottic view using the videolaryngoscopes compared to direct laryngoscopy is unsurprising given that these devices all have a video camera positioned at the end of their blades. However, an improved view with use of the CMAC compared with use of the Storz VL deserves some discussion, as at first glance the devices appear very similar. However, on closer inspection the Storz VL camera (fiberoptic bundle based device) is located nearer to the tip of the blade resulting in a slightly limited view compared to the CMAC. Subject feedback provided after the intubation attempts suggested that the view provided by the Storz VL is more easily obscured during the passage of the tube through the pharynx, resulting in transient loss of glottic view. At present there is nothing in the literature comparing the glottic view obtained when using these two laryngoscopic devices. This feature of blade design has not been reported in the literature but may be of use in the design of future videolaryngoscopes. An optimal camera position must exist along the blade where a good view of laryngeal inlet can be obtained and the process of successful passage of the endotracheal tube observed.

The Glidescope and Storz videolaryngoscopes took significantly longer to intubate the manikin trachea compared to both the Macintosh blade and CMAC. A longer time to intubation when using the Glidescope and other video laryngoscopes is well reported in the literature in both manikins and patients. This finding is significant, as the subjects had the most clinical experience with the Glidescope before testing on the manikin, implying that this is unlikely to be due to a learning effect associated with this device. No difference in TTSI was found between the use of the Macintosh blade and CMAC. Presently there is little published work on the performance characteristics of the CMAC video laryngoscope but median time to intubation value of 19 seconds found the current study is very similar to that found in the only published human case series of CMAC use which found an average of 16 seconds to intubation [22]. This finding is significant as it was the device that subjects had the least experience with before the study. The reason for the relatively short time to intubate associated with use of the CMAC may be related to the similarity of its use with the known skill of direct laryngoscopy, the position of the camera on the blade allowing a view of the process tube passage into the trachea and the relatively direct route of ETT passage through the upper airway. Despite the expected improvement in glottic view associated with the use of the videolaryngoscopy the Macintosh blade maintained a high rate of success and short time to intubation. These findings reflect the well-established efficacy and subject experience of this device in day to day anesthetic practice.

The subjects had clinical experience of the Macintosh laryngoscope and some limited experience with use of the Glidescope before testing. They had no experience with use of the Storz Video Laryngoscope or the CMAC. This is a limitation of the study, but did not result in a performance advantage in use of the Glidescope, but may have contributed to the poor performance associated with use of the Storz Video Laryngoscope.

The gradual improvement in performance associated with the performance of a similar task that of laryngoscopy in this instance must be accounted for in the methodology [23] where repeated measures are made on the same subject. It was found that for all the methods of laryngoscopy the practice effect was weak during the task as a whole, as assessed by the low r-value of correlation amongst try in the GEE and GLM models.

The use of an airway manikin instead of patients when investigating airway devices results in limited generalizability. The manikin will only reproduce one aspect of a difficult airway (limited neck extension and poor glottis view in this case). A high level of caution must be applied if any of the findings are to be extrapolated to a human difficult airway population. Despite these shortcomings and reservations, we felt the high fidelity airway manikin produced many attributes of a difficult laryngoscopic view of the human glottis. Additionally, the study at laryngoscopic subjects suggested the airway scenario looked and felt realistic.

## Conclusion

During a manikin simulation of difficult laryngoscopy use of all the video laryngoscopes resulted in a better glottic view than the Macintosh blade. Specifically, the CMAC was found to provide a better laryngoscopic view than the Storz videolaryngoscope. Use of the Glidescope or Storz videolaryngoscope resulted in a prolonged time to successful intubation compared with use of either the CMAC or Macintosh blade. The features of the CMAC and Macintosh blade, revealed in this manikin study, suggest their use combines efficacy with expediency.

## Competing interests

Paul Picton is a consultant for Medtronic ENT Division.

The other authors had no competing interests in the performance of this study.

## Authors’ contributions

DH designed the study, tested the subjects, analyzed the data, and composed the manuscript. PP helped in the study design, tested the subjects, participated in the data analysis, and contibuted to the manuscript. MM provided statistical advice and composed the graphs. CT participated in the study design and coordinated the team efforts. All authors read and approved the final manuscript.

## Pre-publication history

The pre-publication history for this paper can be accessed here:

http://www.biomedcentral.com/1471-2253/12/11/prepub
